# β-(1→3)-D-glucan modulates DNA binding of nuclear factors κB, AT and IL-6 leading to an anti-inflammatory shift of the IL-1β/IL-1 receptor antagonist ratio

**DOI:** 10.1186/1471-2172-7-5

**Published:** 2006-03-22

**Authors:** Juergen Luhm, Ulrich Langenkamp, Jenny Hensel, Christoph Frohn, Joerg M Brand, Holger Hennig, Lothar Rink, Petra Koritke, Nadine Wittkopf, David L Williams, Antje Mueller

**Affiliations:** 1 Institute of Immunology and Transfusion Medicine, Medical School, University of Lübeck, Germany; 2 Department of Surgery and Immunopharmacology Research Group, East Tennessee State University, Johnson City, USA; 3 Department of Rheumatology, University Hospital of Schleswig-Holstein, Campus Lübeck, Germany

## Abstract

**Background:**

β-1→3-D-glucans represent a pathogen-associated molecular pattern and are able to modify biological responses. Employing a comprehensive methodological approach, the aim of our *in vitro *study was to elucidate novel molecular and cellular mechanisms of human peripheral blood immune cells mediated by a fungal β-1→3-D-glucan, *i.e*. glucan phosphate, in the presence of lipopolysaccharide (LPS) or toxic shock syndrome toxin 1 (TSST-1).

**Results:**

Despite an activation of nuclear factor (NF)κB, NFinterleukin(IL)-6 and NFAT similar to LPS or TSST-1, we observed no significant production of IL-1β, IL-6, tumor necrosis factor α or interferon γ induced by glucan phosphate. Glucan phosphate-treated leukocytes induced a substantial amount of IL-8 (peak at 18 h: 5000 pg/ml), likely due to binding of NFκB to a consensus site in the IL-8 promoter. An increase in IL-1receptor antagonist(RA) production (peak at 24 h: 12000 pg/ml) by glucan phosphate-treated cells positively correlated with IL-8 levels. Glucan phosphate induced significant binding to a known NFIL-6 site and a new NFAT site within the IL-1RA promoter, which was confirmed by inhibition experiments. When applied in combination with either LPS or TSST-1 at the same time points, we detected that glucan phosphate elevated the LPS- and the TSST-1-induced DNA binding of NFκB, NFIL-6 and NFAT, leading to a synergistic increase of IL-1RA. Further, glucan phosphate modulated the TSST-1-induced inflammatory response via reduction of IL-1β and IL-6. As a consequence, glucan phosphate shifted the TSST-1-induced IL-1β/IL-1RA ratio towards an anti-inflammatory phenotype. Subsequently, glucan phosphate decreased the TSST-1-induced, IL-1-dependent production of IL-2.

**Conclusion:**

Thus, β-1→3-D-glucans may induce beneficial effects in the presence of pro-inflammatory responses, downstream of receptor binding and signaling by switching a pro- to an anti-inflammatory IL-1RA-mediated reaction. Our results also offer new insights into the complex regulation of the IL-1RA gene, which can be modulated by a β-1→3-D-glucan.

## Background

β-1→3-D-glucans occur as a principal component of microbial cell walls or can be secreted from both, non-pathogenic and pathogenic fungi such as *S. cerevisae *and *C. albicans *[1]. These β-1→3-D-linked glucose polymers are characterized as a fungal pathogen-associated molecular pattern (PAMP) [2]. The primary cellular recognition of β-1→3-D-glucans is mediated by several β-1→3-D-glucan receptors on phagocytes [3,4] and other cells [5,6]. Human as well as murine Dectin-1 has been demonstrated to be the major pattern recognition receptor (PRR) for intact yeast and β-1→3-D-glucan-containing particles (*i.e*. zymosan) on monocytes/macrophages as well as neutrophils and on primary cells [7-11]. In the murine system, binding of zymosan to Dectin-1 resulted in production of TNFα through Toll-like receptor 2 and the adaptor protein MyD88 [12]. Another water-soluble β-1→3-D-glucan (PGG-glucan) has been described to activate NFκB and NFIL-6 in murine cell lines [13,14]. Similarly, it has been shown that β-1→3-D-glucans activate NFκB in a human monocyte-like cell line [15] and in human polymorphonuclear neutrophils (PMN), in the latter case without secretion of pro-inflammatory cytokines (IL-1, IL-6, TNFα) [16]. One study proposed that the production of the anti-inflammatory IL-1RA, but not IL-1 by human monocytes may be a potentially protective mechanism induced by β-1→3-D-glucan [17]. Three other investigations have reported that human leukocytes and human vascular endothelial cells produce IL-8 in response to zymosan [18] or a water-soluble β-1→3-D-glucan [6,19].

In addition, β-1→3-D-glucans seem to be able to modify the response to pro-inflammatory stimuli or even sepsis. In a murine polymicrobial sepsis model, β-1→3-D-glucan [20] treatment resulted in decreased septic morbidity and mortality mediated via inhibition of NFκB and stimulation of the phosphoinositide-3-kinase (PI3K) pathway [21,22]. These and other animal studies [23,24] as well as a clinical trial [25] support a protective role of β-1→3-D-glucan in certain pro-inflammatory conditions. The mechanisms underlying these beneficial effects of β-1→3-D-glucan are only partially resolved, especially in humans. Thus, the aim of this study was to elucidate molecular and cellular mechanisms of β-1→3-D-glucans on human leukocytes in pro-inflammatory conditions with special emphasis on the cytokine profile and its transcriptional regulation. For this purpose, peripheral blood mononuclear cells (PBMC) were exposed to a well-defined β-1→3-D-glucan, *i.e*. glucan phosphate (GP) [20,26], alone or simultaneously with LPS from gram-negative bacteria or the superantigen TSST-1 from gram-positive bacteria over 48 h. Because of the potential effect of β-1→3-D-glucan on cytokine production [12,16-19], TNFα, IL-1β, IL-6, IL-8 and IL-1RA were measured as well as IFNγ, IL-2, IL-4, IL-10, IL-12 and TGFβ1. Correspondingly, four NFκB sites from the TNFα promoter (κ consensus, κ1, κ2, κ3) [27], a κ consensus site from the IL-8 promoter [28], an NFAT site from the IFNγ promoter (ATP2) [29] and a consensus NFIL-6 site from the IL-6 promoter [13] were examined. Because of the anti-inflammatory role of IL-1RA, we focused on binding of transcription factors to the IL-1RA promoter. An inhibitory element and three positive-acting LPS-response elements (LRE-1, LRE-2 and LRE-3) in the IL-1RA promoter, including NFκB, PU.1 and NFIL-6 sites, have been characterized previously [30-33]. Using computational analysis for homology search [34], we looked for new binding motifs in the IL-1RA promoter.

## Results

### Binding activities of NFκB, NFIL-6 and NFAT from human PBMC to the TNFα, IL-6 and IFNγ promoters following an *in vitro *stimulation (1 h) with LPS, TSST-1, GP, GP + LPS and GP + TSST-1

**Figure 1:**
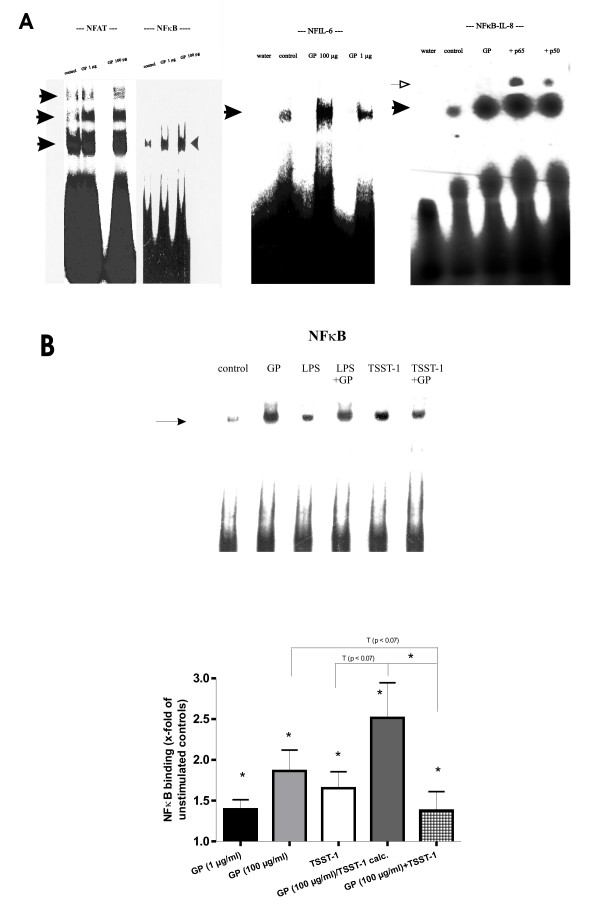
**A, GP led to DNA binding of NFAT, NFκB and NFIL-6. **Human PBMC were incubated with medium control or GP (1 and 100 μg) for 1 h at 37°C. Nuclear extracts were incubated with a ^32^P-labeled NFAT, NFκB, NFIL-6 or NFκB-IL-8 oligonucleotide probe corresponding to the IFNγ, TNFα, IL-6 and IL-8 gene promoters (Table 1). Arrows with the black head indicate migrational location of the NFAT-DNA, NFκB-DNA, NFIL-6-DNA or NFκB-IL-8-DNA complex compared to free probe (no shift). Arrow with the open head indicates a supershift of NFκB-IL-8-DNA-anti-p65 and -p50, respectively. An autoradiogram from a representative experiment is shown (n = 7). **B, Decreased DNA binding of NFκB following GP + TSST-1, compared to TSST-1, GP, or TSST-1/GP. **Human PBMC were incubated with medium control, LPS, TSST-1, GP (100 μg), GP + TSST-1 and GP + LPS for 1 h at 37°C. An autoradiogram from a representative experiment is shown. Schematic representation of NFκB-DNA binding activity following GP (1 and 100 μg) or TSST-1 treatment (250 ng) as well as simultaneous administration of both compounds and the theoretical value (GP/TSST-1 calc.). Medium control levels were set equal to 1± SEM and significances (* = p < 0.05) are shown with respect to medium control (n = 4).

**Figure 2:**
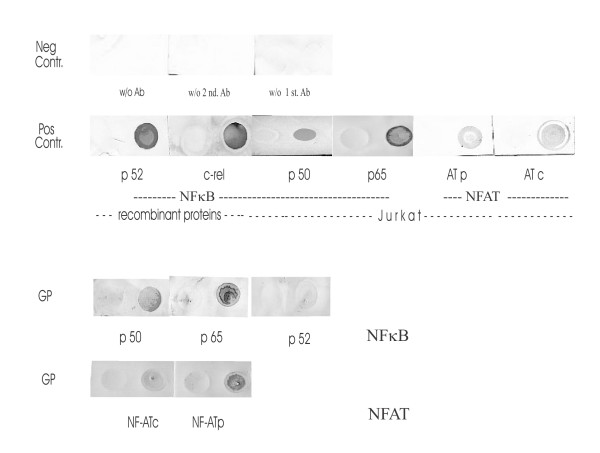
**NFκB p65/p50 and NFATc2/c1 were involved in GP-induced DNA binding. **In order to determine whether the DNA binding proteins activated by GP were related to NFκB and NFAT, an immuno(dot) blot was performed. A representative dot blot showing staining of negative and positive controls (recombinant proteins NFκB p52, c-rel; nuclear extracts of PMA-treated Jurkat cells positive for NFκB p50, p65 and for NFATc1, c2) in the upper panel and staining of nuclear extracts of GP-treated PBMC (positive for NFκB p65, p50 and NFAT c2, c1) in the lower panel is shown (n = 3).

GP induced band shifts, indicating binding of NFκB as well as of NFIL-6 to the corresponding DNA oligonucleotides (κ consensus, κ1, κ2, κ3 sites from the TNFα promoter; κ consensus from the IL-8 promoter; NFIL-6consensus from the IL-6 promoter; Table 1; Fig. 1A). The extent of the band shifts induced by GP was not statistically different when compared to LPS or TSST-1 (Fig. 1B). A supershift, conducted for an NFκB consensus site from the IL-8 promoter, demonstrated a GP-induced predominant binding of NFκB p65 and to a lesser degree of p50 (Fig. 1A). Accordingly, an immunoblot of nuclear extracts from GP-treated PBMC showed a strong binding of NFκB p65 and a weaker reaction of p50, whereas p52 was negative (Fig. 2). Simultaneous co-treatment of PBMC with GP did not change the LPS-induced NFκB binding to oligos from the TNFα promoter significantly (Fig. 1B), but substantially decreased the TSST-1-induced NFκB binding when compared to TSST-1 or GP (100 μg) and thus differed completely from the theoretical value of GP/TSST-1 calc. (n = 4; p < 0.05 vs. GP/TSST-1 calc. and p = 0.07 vs. TSST-1; Fig. 1B). GP was also able to induce band shifts indicative of binding of particular variants of NFAT to an oligo from the IFNγ promoter (Fig. 1A). An immunoblot for two NFAT subunits demonstrated a GP-induced binding of NFATc2 and to a lesser degree of NFATc1 (Fig. 2).

**Table 1:** Sense strand of oligonucleotides used in EMSAs.

Oligonucleotides^a^	Base sequences (5'- XXX -3')	binding site/cytokine gene promoter (position of the cytokine gene from the TSS)^b^
NFκBcS	GAT CCT CAG AGG GGA CTT TCC^c ^GAT G	NFκB consensus sequence/TNFα promoter [27]
NFκB1S	GAT CCT GGG ACA GCC CAG	NFκB1 sequence/TNFα promoter [27]
NFκB2S	GAT CCG GGG TAT CCT G	NFκB2 sequence/TNFα promoter [27]
NFκB3S	GAT CCT GGG TTT CTC CG	NFκB3 sequence/TNFα promoter [27]
IL-8κBcS	ATC GTG GAA TTT CCT CTG A	NFκB consensus sequence/IL-8 promoter [28]
NFATP2S	GAT CTA AAA TTT CCA GTC CTT GA	NFATP2 sequence/IFNγ promoter [29]
NFIL-6S	TGC AGA TTG CGC AAT CTG CA	NFIL-6 consensus sequence/IL-6 promoter [13]
NFκBcS	GCG AGG AGG GTA TTT CCG CTT	between -80 and -100 NFκB consensus/IL-1RA promoter [30]
NFκB3S	ACA ACA GCA AGG GTT TCT CTT TTT GGA AAT	between -100 and -130 NFκB3/IL-1RA promoter
NFκBcS	AGT AGG GAG TTT GGT	between -266 and -280 NFκB consensus sequence/IL-1RA promoter
NFκB2/3S	ACT CTG GGT ACC TGT	between -288 and -302 NFκB2/3 sequence/IL-1RA promoter
NFATP2/3S	GGC GCA CAA AAC CTA AAA TAT TTA CTA TCT	between -471 and -500 NFATP2/3 sequence/IL-1RA promoter
NFIL-6S	TTA CAA CAC TCC ATT GCG ACA CTT AGT GGG	between -140 and -170 NFIL-6 sequence/IL-1RA promoter [31]
Oct-1 DNAS	AAT TGC ATT GCC TGC AGG TCG ACT CTA GAG GAT CCA TGC AAA TGG ATC CCC GGG TAC CGA GCT C	exclusion of unspecific bindings, unrelated DNA, (Amersham Pharmacia)

(**a: **S = sense; **b: **TSS = transcription start site;**c: **___ = main binding motif).

### Cytokine profile of human PBMC following a 48 h *in vitro *stimulation with LPS, TSST-1, GP, GP + LPS and GP + TSST-1

Because of the GP-induced band shifts to seven sites from the TNFα, IL-8, IL-6 and IFNγ promoters, it could be assumed that GP treatment of human PBMC would lead to production of TNFα, IL-8, IL-6, IFNγ and other pro-inflammatory mediators. Previous studies reported that β-1→3-D-glucans induced only a limited cytokine secretion of human blood cells [15-18]. Corroborating and extending these singular findings in terms of examining pro- (eight) as well as anti- (three) inflammatory cytokines over time (48 h) and six additional transcription factor binding sites, our comprehensive analysis revealed the following cytokine profile of human leukocytes in response to a highly purified water soluble β-1→3-D-glucan and in comparison to two pro-inflammatory mediators (LPS, TSST-1):

#### (i) IL-1β

**Figure 3:**
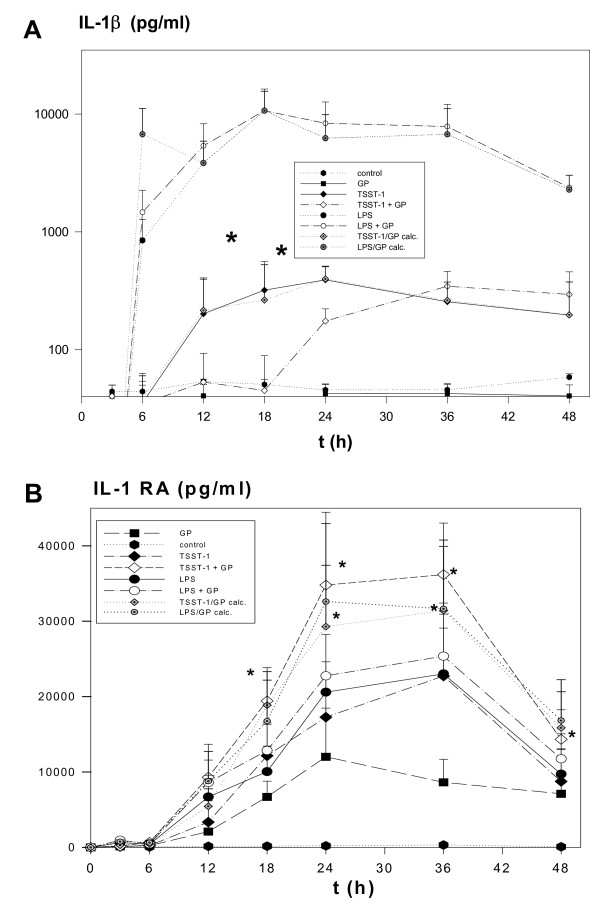
**A, IL-1β was down-regulated (12 h – 24 h) following GP + TSST-1 when compared to TSST-1**. Time course (48 h) of IL-1β production (pg/ml) by human PBMC incubated with medium control, 100 μg GP, 250 ng TSST-1, GP + TSST-1, 250 ng LPS and GP + LPS. At 18 h and 24 h there was a marked reduction in IL-1β production following GP + TSST-1 when compared to TSST-1 (* = p < 0.05). Graphs depicting the theoretical values for GP/LPS calc. and GP/TSST-1 calc. are also shown. IL-1β levels in the supernatnant were obtained by Elisa and are shown as mean ± SEM (n = 3). **B, IL-1RA was exaggerated following GP + TSST-1 (12 h – 48 h) when compared to TSST-1. **Time course (48 h) of IL-1RA production (pg/ml) by human PBMC incubated with medium control, 100 μg GP, 250 ng TSST-1, GP + TSST-1, 250 ng LPS and GP + LPS. Between 18 h and 48 h a simultaneous treatment of PBMC with GP + TSST-1 led to a synergistic effect, *i.e*. a higher IL-1RA production when compared to an addition of the single values for TSST-1 and GP (* = p < 0.05). Graphs depicting the theoretical values for GP/LPS calc. and GP/TSST-1 calc. are included. IL-1RA levels in the supernatnant were obtained by Elisa and are shown as mean ± SEM (n = 3).

There was no IL-1β production detectable following PBMC treatment with 1 nor 100 μg GP. An insignificant up-regulation of IL-1β production for GP + LPS was observed in comparison with LPS. For GP + TSST-1 we found a significant reduction in IL-1β from 18 h – 24 h, for the latter by about 40% when compared to TSST-1 or the theoretical value of GP/TSST-1 calc. (n = 6; both p = 0.01). On the whole, GP mediated a reduction of the TSST-1-induced amount of IL-1β by about 50% (Fig. 3A).

#### (ii) IL-6

GP induced only a small amount of IL-6 and there was no significant alteration of the LPS-induced IL-6 production by GP. On the contrary, we observed a decreased IL-6 production for GP + TSST-1 when compared to TSST-1 or GP/TSST-1 calc., especially at 24 h by about 40% (n = 5; both p = 0.02), (data not shown in detail).

#### (iii) IL-8

We found that GP induced a substantial IL-8 production, in comparison with medium control, especially at 24 h (n = 13; p < 0.01). A combination of GP and LPS resulted in a non-significant increase of IL-8 production when compared to LPS or GP/LPS calc. There was no significant alteration in IL-8 production following GP + TSST-1 when compared to TSST-1 or GP/TSST-1 calc. (data not shown in detail).

#### (iv) IL-1RA

Besides IL-8, IL-1RA was the only mediator which was produced in significant quantities following GP treatment, especially at 24 h (n = 14, p < 0.01). When compared to LPS stimulation or GP/LPS calc., GP + LPS did not alter the kinetic course of the IL-1RA production. However, following GP + TSST-1 we found a synergistic increase in IL-1RA production from 18 h to 48 h, when compared to TSST-1 (for instance at 24 h: n = 6; p = 0.01) or to the theoretical value of GP/TSST-1 calc. (from 18 h to 36 h). Over the time course of 48 h, GP elevated the TSST-1-induced amount of IL-1RA by approximately 200% (Fig. 3B).

*Positive correlation between GP-induced IL-8 and IL-1RA productions*. Following stimulation with GP (24 h), we observed a positive correlation between IL-8 and IL-1RA. Moreover, we found this correlation to be dose-dependent, since 100 μg of GP induced larger amounts of IL-8 and IL-1RA than 1 μg GP (n = 5, p ≤ 0.002, r = 0.9 and 0.98, respectively).

#### (v) TNFα

With respect to TNFα production, 100 μg of GP yielded minor, statistically not distinguishable amounts, when compared to medium control. GP + LPS did not change the TNFα production when compared to LPS supplementation or GP/LPS calc. When combined with TSST-1 or GP/TSST-1 calc., GP seemed to exert a synergistic effect on TNFα secretion after 36 h (n = 3; p < 0.05), but overall an enhancement of only 10% was observed (p > 0.05; data not shown in detail).

#### (vi) IFNγ

No IFNγ production was detectable following treatment of PBMC with 100 μg GP. There was a minor increase in IFNγ production following GP + LPS at 36 h, when compared to LPS or GP/LPS calc., and a slight down-regulation following GP + TSST-1 at 24 h and 36 h, when compared to TSST-1 or GP/TSST-1 calc. (data not shown).

#### (vii) IL-2, IL-12, IL-4, IL-10, TGFβ1

The production of IL-2, IL-12, IL-4, IL-10 and TGFβ 1 was not induced by GP. There were also no significant differences between medium control, LPS, TSST-1, GP, GP + LPS, GP/LPS calc. and GP + TSST-1 or GP/TSST-1 calc. over 48 h (data not shown).

### Role of NFκB, NFIL-6 and NFAT binding in LPS-, TSST-1-, GP-, GP + LPS- and GP + TSST-1-induced IL-1RA expression by human PBMC

**Figure 4:**
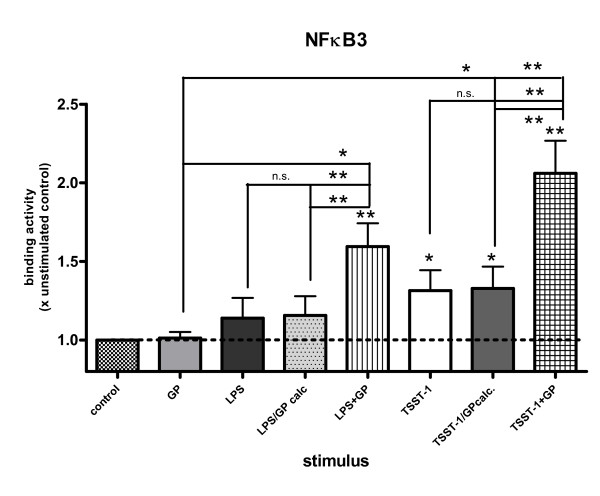
**GP modulated LPS- and TSST-1-induced binding to a new NFκB3 site in the IL-1RA promoter. **Binding was enhanced for GP + LPS and GP + TSST-1, when compared to LPS, TSST-1, GP, GP/LPS calc. or GP/TSST-1 calc., respectively (* = p < 0.05; ** = p < 0.01). Medium control levels were set equal to 1 ± SEM (n = 6). DNA binding was assessed by EMSA (see methods).

**Figure 5:**
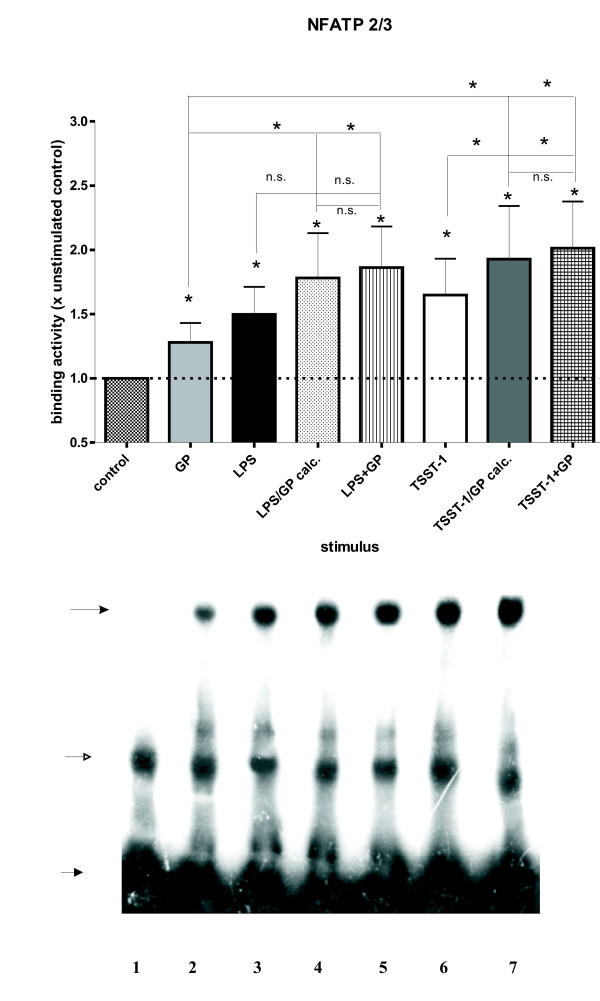
**GP modulated TSST-1-induced DNA binding to a new NFATP2/3 site in the IL-1RA promoter. **Binding was up-regulated for GP + LPS and GP + TSST-1, when compared to GP and, in the latter case, to both GP and TSST-1 (* = p < 0.05). Human PBMC were incubated with medium control, LPS, TSST-1, GP, GP + TSST-1 and GP + LPS for 1 h at 37°C. Schematic representation of the elevated NFAT-DNA binding activity to the site in the IL-1RA promoter. Bars depicting the theoretical values for GP/LPS calc. and GP/TSST-1 calc. are added. Medium control levels were set equal to 1 ± SEM (n = 6). An autoradiogram of a representative experiment is shown in the lower panel (lane 1: water; lane 2: medium control; lane 3: GP; lane 4: LPS; lane 5: TSST-1, lane 6: GP + LPS; lane 7: GP + TSST-1).

Because GP induced band shifts to NFκB, NFIL-6 and NFAT sites, but no TNFα, IL-6 or IFNγ, we hypothesized that the activated transcription factors might bind to sites in the IL-1RA promoter. We investigated previoulsy described and newly discovered transcription factor binding sites of the IL-1RA promoter (Table 1) using the program OMIGA (v.1.1.3, Oxford Molecular, Oxford, UK). The LPS responsive κB consensus site of LRE-1 (between -84 and -93) [30] displayed band shifts, but no significant alterations of its binding activity, irrespective of the treatment (data not shown). A new κB3 site (-100 and -130) within the LRE-1 [30] showed increased binding induced by TSST-1 (+30% vs. control), but GP co-treatment up-regulated the LPS (+65% vs. control)- and the TSST-1 (+110% vs. control)-induced binding in a superadditive synergistic fashion, when compared to medium control as well as to LPS, TSST-1 or GP or the theoretical values of GP/LPS calc. or GP/TSST-1 calc. (all p < 0.05; Fig. 4). Binding to a new κB consensus site (between -266 and -280) was significantly elevated for GP + TSST-1 (p < 0.05; data not shown). The LPS-, TSST-1- and GP + LPS-induced binding to the new κB2/3 site (-288 and -302) was down-regulated by about 20–35% in comparison with medium control (p < 0.05; data not shown). The NFIL-6 site (-147 and -170) close to LRE-2 [31,32] showed an up-regulation of binding for all compounds and combinations vs. medium control, ranging from 25–110% (p < 0.05). Further, GP + TSST-1 induced an increased binding to the NFIL-6 site in comparison with TSST-1 (1.3fold) or GP (1.6fold; p < 0.05), (data not shown). A new binding site for NFATP2/3 was found more distal of the transcription start site, between -471 and -490, displaying again a substantial increase of binding for all compounds and combinations, when compared to medium control (p < 0.05). In addition, binding to the NFATP2/3 site following both, GP + LPS (1.4fold of GP) and GP + TSST-1 (1.5fold of GP, 1.25fold of TSST-1) was elevated (p < 0.05; Fig. 5), but only in an additive synergistic manner, *i.e*. the experimental results were not different from the theoretical values (calc.).

### GP modulated the TSST-1-induced IL-1β/IL-1RA ratio towards an anti-inflammatory phenotype

**Figure 6:**
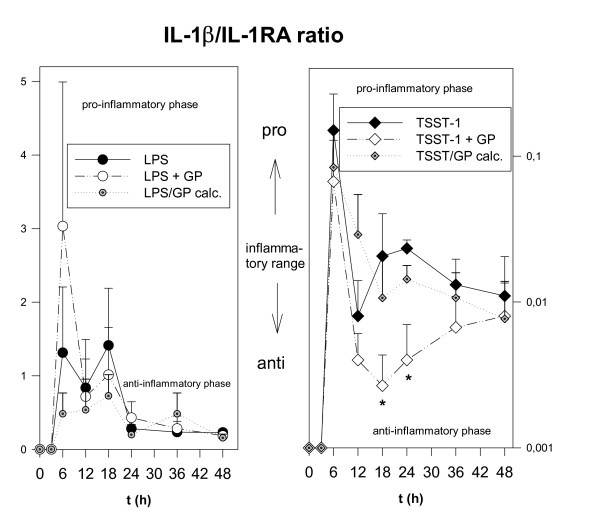
**Decreased IL-1β/IL-1RA ratio following GP + TSST-1 when compared to TSST-1. **Time course (48 h) of the IL-1β/IL-1RA ratio following incubation of human PBMC with LPS, TSST-1, GP + LPS, GP/LPS calc., GP + TSST-1 or GP/TSST-1 calc. LPS and GP + LPS treatments resulted in generating more IL-1β than IL-1RA up to 24 h. When comparing LPS or GP/LPS calc. vs GP + LPS we did not find significant alterations in the IL-1β/IL-1RA ratio over 48 h. With respect to TSST-1 or GP/TSST-1 calc. vs GP + TSST-1 we observed a pronounced anti-inflammatory action of GP, as demonstrated by an about 10–100 fold reduced ratio at 18 h and 24 h, *i.e*. a higher production of IL-1RA than of IL-1β (* = p < 0.05). Data are shown as mean ± SEM (n = 3).

In a simplified approach, IL-1β and IL-1RA might represent two sides of a coin, *i.e*. pro- or anti-inflammatory action. Therefore, we applied the IL-1β/IL-1RA ratio as an indicator of the degree of inflammation. Both, LPS and GP + LPS treatments resulted in generating more IL-1β than IL-1RA at early time points, *i.e*. up to 24 h. Comparing LPS vs. GP + LPS, we did not find substantial alterations over a time course of 48 h in the IL-1β/IL-1RA ratio, only a – non-significant – 2.5fold higher ratio at 6 h. With respect to TSST-1 or the theoretical value of GP/TSST-1 calc. vs. GP + TSST-1, we observed a pronounced anti-inflammatory action of GP, as demonstrated by an about 10–100 fold reduced ratio at 18 h and 24 h of incubation, *i.e*. a higher production of IL-1RA than of IL-1β (n = 3; both p < 0.05; Fig. 6). A biological relevance of this result was suggested by a decrease of the IL-1 dependent release of IL-2 from murine EL-4 cells following GP + TSST-1 when compared to TSST-1 or GP/TSST-1 calc. (data not shown in detail, n = 4; p < 0.07).

### Inhibition of transcription factors led to reduced IL-1RA levels

**Figure 7:**
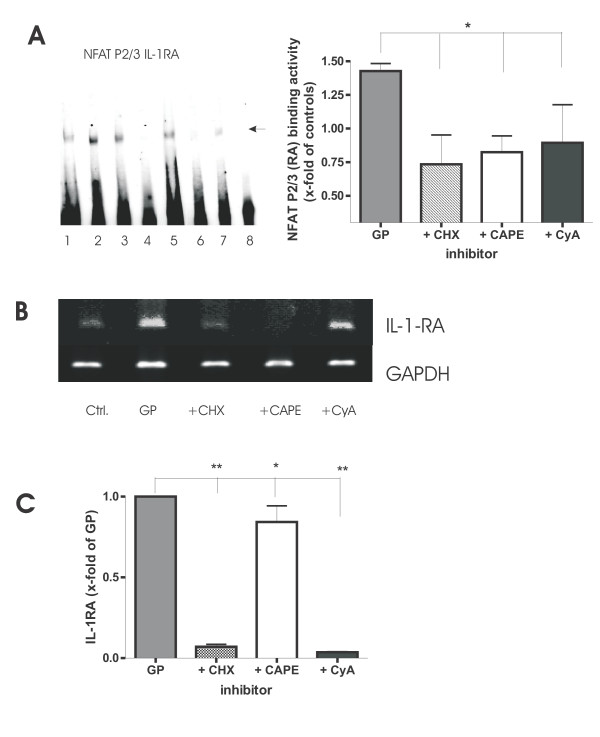
**Inhibition of GP-induced NFκB, NFAT and NFIL-6 DNA binding to the IL-1RA promoter. A, **A representative autoradiogram (lane 1: control, lane 2: GP, lane 3: GP + unlabeled mutated NFATP2/3 oligo, lane 4: GP + CAPE, lane 5: GP + CyA, lane 6: GP + CHX, lane 7: GP + unlabeled NFATP2/3 oligo, lane 8: water) as well as a graphical summary for the NFATP2/3 site, depicting a decrease in binding following inhibition when compared to GP is displayed (* = p < 0.05). Data are shown as mean ± SEM (n = 4). **B**, Inhibition of NFκB, NFAT and NFIL-6 resulted in decreased IL-1RA mRNA following CHX, CAPE or CyA compared to GP. GAPDH is used as housekeeping control (representative gel, n = 4). **C**, Inhibition of NFκB, NFAT and NFIL-6 significantly reduced the production of IL-1RA by human PBMC following treatment with CHX, CAPE or CyA when compared to GP (* = p < 0.05; ** = p < 0.01). Data are shown as mean ± SEM (n = 3).

Pharmacological inhibitors were used to demonstrate that the transcription factor sites within the IL-1RA promoter are relevant for the induction of IL-1RA by GP. As expected, an inhibition of NFκB via CAPE [35] and CyA, NFAT via CyA and NFIL-6 via CHX [36] could not be overruled by GP, leading to a down-regulated binding activity of the transcription factors to the IL-1RA sites, and a reduction of IL-1RA mRNA and IL-1RA protein levels (Fig. 7). The extent of reduction in NFATP2/3 binding is exemplarily shown in an autoradiogram (Fig. 7A, left side) and graphically summarized (n = 4, Fig. 7A, right side). The results for the NFκB and NFIL-6 binding sites were similar (data not shown). Because the highest IL-1RA amount was found at 24 h, mRNA was examined following 1 h inhibition and 18 h of GP. A representative gel demonstrating a decrease in IL-1RA mRNA after inhibition with CAPE, CyA and CHX is displayed in Fig. 7B (n = 4). In addition, corresponding IL-1RA protein levels after 24 h of GP are shown in Fig. 7C (n = 3). Altogether, NFκB inhibition by CAPE was more pronounced at the binding activity and the transcription, whereas CyA and CHX mainly led to a decreased IL-1RA release.

## Discussion

Animal models of sepsis and myocardial injury suggest that a β-1→3-D-glucan like GP mediates its protective mechanisms, in part, via a rapid shift from NFκB- to PI3K-signaling [22,37]. Our study provides novel evidence that downstream of recognition and signaling pro-inflammatory transcription factor binding and cytokine expression of human leukocytes is switched to an anti-inflammatory phenotype by GP.

**Figure 8:**
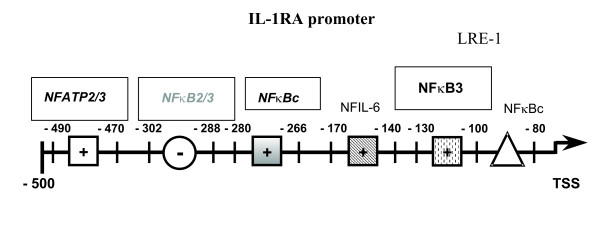
**Cartoon of the sense strand of the IL-1RA promoter. **Relative locations of previously (LRE-1) [30-33] and newly described (boxed), positively (black) and negatively (gray) regulatory transcription factor binding sites are depicted.

We confirmed and extended previous results [13-16] indicating that, in the absence of other stimuli, β-1→3-D-glucans induced binding of NFκB-, NFIL-6- and NFAT-mers to cytokine promoters. Because of the multiple band shifts observed for NFAT binding (Fig. 1A), one could speculate that there is activation of several different NFAT isoforms, probably derived from alternative splicing [38]. Interestingly, the GP-induced transcription factor binding transformed only into a very limited cytokine response, namely IL-8 and IL-1RA (Fig. 3B). Hence, our data are in aggreement with the few reports describing a β-1→3-D-glucan-mediated IL-8 [6,16,19] and IL-1RA production [17]. In addition, our EMSA/supershift and immunoblotting results demonstrated a GP-mediated predominant binding of NFκB p65 and to a lesser extent of p50 to a κB consensus site of the IL-8 promoter (Figs. 1A, 2). Results by Schulte and colleagues [28] pointed to an induction of IL-8 transcription depending on activation via an NFκB p65/65 homodimer, rather than via p65/50 heterodimers, which might be the case for the GP-mediated IL-8 transcription. A GP-mediated IL-8 transcription based upon a cooperation between transactivated NFκB p65 and NFIL-6 [13,39] or NFATc2 dimer binding to the IL-8κB site [38] seems also possible. The IL-8κB consensus site exhibits a preferentially p65 binding half site and thus differs from the κB half site described for TNFα and IL-1β [27,40], supporting the idea of regulating NFκB binding through combinatorial associations of the subunits and the specific sequence of the decameric κB motif [41,42]. Unlike LPS or TSST-1, we found that GP did not induce IL-1β, but it strongly induced IL-1RA, suggesting an immediate anti-inflammatory potential of GP. Analysing the IL-1RA promoter [34], we discovered four new binding sites (Fig. 8): an NFκB3 site (between -100 and -130), an NFκB consensus site (-266 and -280), another NFκB2/3 site (-288 and -302) and a more distal NFATP2/3 site (-471 and -490). Our data indicated that GP leads to production of IL-1RA primarily via induction of NFATP2/3 and NFIL-6 DNA binding, which might be due to differences in the binding motif or the composition of the activated transcription factors (NFAT) between the IFNγ, IL-6 and IL-1RA promoter. The differential decrease of NFκB, NFAT and NFIL-6 binding to sites in the IL-1RA promoter as well as of IL-1RA mRNA and protein induced by selective inhibitors prior to GP treatment might suggest that these steps are linked to each other and necessary for induction of IL-1RA (Fig. 7). Regarding cellular sources, both, monocytes as well as neutrophils have been reported to produce IL-1RA [32]. Flow cytometric experiments seemed to confirm that monocytes and neutrophils were able to produce IL-8 just as IL-1RA in response to GP (data not shown). This GP-induced cytokine profile was substantially more restricted than that of LPS or TSST-1, which is likely due to differences in recognition and signaling between LPS, TSST-1 and GP. Recognition of LPS is mainly mediated through Toll-like receptor 4 and subsequent signaling via the NFκB pathway, leading to expression of pro-inflammatory mediators like TNFα [43]. A bacterial superantigen like TSST-1 acts through binding to MHC-II molecules and subsequently the T cell receptor, leading to release of mainly IFNγ and TNFα, the latter via both, PI3K and p38 mitogen-activated kinase signaling [44,45]. Altogether, the induction of the neutrophil-attracting IL-8 and the anti-inflammatory IL-1RA by fungal carbohydrates (GP) may well fit to a benign PAMP response, mounting defensive mechanisms against a possible microbial attack.

Potentially even more relevant than the specific cytokine panel induced by GP, we found that, in the presence of other stimuli (LPS or TSST-1), GP ameliorated their pro-inflammatory immune reactions, similar to the effects in murine models of sepsis and inflammation [21-24]. Mainly, GP altered the TSST-1-induced IL-1β/IL-1RA ratio from a pro- to an anti-inflammatory profile via down-regulation of IL-1β and IL-6, at the same time there was a synergistic up-regulation of IL-1RA. In part, this change may be caused by GP-mediated modulations of the TSST-1 induced binding of NFκB, NFIL-6 and NFAT to known and up to now unknown sites within the IL-8 and IL-1RA promoter instead of the TNFα, IL-1β, IL-6 or IFNγ promoter. Accordingly, we found that the TSST-1-induced binding of NFκB to sites from the TNFα promoter negatively correlated with the GP-mediated enhancement of the TSST-1-induced IL-1RA production (r = -0.88; p < 0.01; data not shown in detail). Of the four examined NFκB sites from the IL-1RA promoter, mostly the TSST-1- and LPS-induced binding to the NFκB3 and the TSST-1-induced binding to the new NFκB consensus site seemed to be increased by GP. So, while we observed a GP-mediated down-regulation of the LPS- and the TSST-1-induced NFκB binding to sites of the TNFα promoter, there was an up-regulation to NFκB3 and NFκB consensus sites of the IL-1RA promoter. These seemingly contradictory data could be explained by differences in either NFκB subunits or conserved nucleotides (#1, 2, 3, 10) within the decameric NFκB binding motif between the TNFα and the IL-1RA promoter (for NFκB3 the IL-1RA motif contains a T on position 10 instead of the conserved C in the TNFα motif, see Table 1), probably leading to differences in binding [41,42]. Despite the location of the new NFκB consensus site (-266 and -280) in the inhibitory element (-250 and -294) of the IL-1RA promoter [30], we observed no inhibition. On the other side, we found an inhibitory NFκB2/3 site (-288 and -302) towards the end of the inhibitory element, demonstrating down-regulations of the LPS- and TSST-1-induced binding, which could not be altered by GP. In our opinion, this site may therefore represent at least a part of the previoulsy described inhibitory element [30]. The GP-modulated increase in TSST-1-induced binding to the new NFκB3 and NFκB consensus site, the NFIL-6 site [32] as well as to the novel NFATP2/3 site may explain the synergistic up-regulation of the TSST-1-induced IL-1RA production. We think that this GP-modulated activation of transcription was reflected by the decrease of the IL-1β/IL-1RA ratio following GP + TSST-1 (Fig. 6). In this context, it has been postulated that *in vitro *a 100fold excess of IL-1RA over IL-1β might control the biological effects of IL-1 [46,47]. Since, in fact, the IL-1β/IL-1RA ratio following GP + TSST-1 is partially less than 0.01, it is not unreasonable to assume that IL-1β bioactivity is inactivated in our system. Indeed, GP reduced the TSST-1-induced, IL-1-dependent IL-2 production of murine EL-4 cells (data not shown in detail).

The weaker modulating effects of GP on the LPS-induced immune response observed in this study, may be attributed to delicately balanced differences in signaling pathways between LPS and TSST-1 [43-45]. TSST-1 has been shown to use the PI3K pathway for signaling [44] and this effect may be sustained by GP treatment [22,37]. It has been demonstrated that in septic/LPS-adapted leukocytes the PI3K pathway selectively controls sIL-1RA but not IL-1β production [48]. Signaling via PI3K has been reported to be involved in the activation of NFAT in T cells [49]. Activation of NFκB can also take place via PI3K [50], which may offer an explanation for a difference in signaling between GP (PI3K) and LPS (mitogen-activated kinase signaling). This idea may be supported by another study showing that despite the use of similar PRR, LPS and peptidoglycan activated the IL-1RA gene through different mechanisms/DNA-binding proteins and acted synergistically in combination, suggestive of signals which are not equivalent in all parts [51].

## Conclusion

In summary, our data demonstrated that *in vitro *glucan phosphate induced a transcription factor binding and a subsequent cytokine profile different from LPS and also from TSST-1 and, moreover, switched a pro-inflammatory TSST-1-induced response to an IL-1RA-mediated anti-inflammatory reaction. Our results also generated new insights into a very complex interplay of transcription factor binding to various known and newly identified sites in the IL-1RA promoter (Fig. 8), which can be regulated differentially by a fungal carbohydrate. Together with the *in vivo *studies [22,37], our findings might support the concept of protective effects mediated by glucan phosphate in pro-inflammatory conditions, especially with a dysregulated IL-1β/IL-1RA ratio [52,53].

## Methods

### Materials

Water soluble phosphorylated β-1→3-D-glucan (GP) was prepared as done before [20]. The physicochemical characteristics of GP were determined as reported previously [20,26]. GP was dissolved in aqueous media, filter-sterilized (0.2 μm) and screened for endotoxin contamination with the Endospecy assay (Seigakaku, Tokyo, Japan), which is specific for endotoxin but does not respond to β-1→3-D-glucans.

### Isolation of human PBMC

Buffy coats were obtained from healthy blood donors in compliance with the Helsinki declaration and with the approval of the ethics committee of the University of Luebeck. The buffy coats were separated over a Ficoll-Hypaque (Biochrom, Berlin, Germany) gradient [54,55]. Following isolation, PBMC were cultured at a final concentration of 3 × 10^6 ^cells/ml (for ELISA measurements) or 5 × 10^6^/ml (for gel shifts) or 1 × 10^6 ^cells/ml (for flow cytometry) in RPMI 1640 medium (BioWhittaker, Heidelberg, Germany, LPS-free), containing 10% heat-inactivated fetal calf serum (FCS, low-LPS, Invitrogen, Karlsruhe, Germany), 1% penicillin (10000 U/ml), 1% streptomycin (10000 μg/ml), and 1% 200 mM L-glutamine (BioWhittaker).

### Stimulation conditions

For induction of transcription factors and cytokines, PBMC were cultured in a volume of 4 ml in sterile pyrogen-free 6-well culture plates (Falcon, Heidelberg, Germany). Cultures were stimulated for 1 h (EMSA) or from 0 h to 48 h (ELISA) with 1 or 100 μg per 10^6 ^cells of GP, with 250 ng/10^6 ^cells TSST-1 (Toxin Technologies, Sarasota, FL, USA) or with 250 ng/10^6 ^cells wild-type LPS from *Escherichia coli *serotype 0111:B4 (Sigma, Munich, Germany). In costimulatory experiments, PBMC were supplemented simultaneously with a combination of GP and TSST-1 or with a combination of GP and LPS.

### Oligonucleotides (Oligos) and 5'-32-P-Labeling

The complementary double-stranded (ds) oligonucleotides (oligos) from the TNFα, IL-8, IFNγ and IL-6 promoters were synthesised from single stranded (ss) oligos (illustrated in Table 1), (TIB Molbiol, Berlin, Germany) and ^32^P-labeled with 5'γ-P-ATP (3,000 Ci/mmol, Amersham, Braunschweig, Germany) using the Ready-To-Go-Polynucleotide-Kinase Kit (PNK Kit, Pharmacia LKB, Freiburg, Germany) according to the manufacturer's instructions. To test the binding specificity of the oligos, mutated oligos were used as an additional control. Mutations in the P2 site of the IFNγ promoter [29] and in the κ consensus sequence of the TNFα promoter [55] have been reported to interfere with sequences, which seem to be crucial for the binding of NFAT and NFκB proteins, respectively. Afterwards, the ds oligos were purified via gel filtration using Probe Quant G-50 Micro Columns (Pharmacia) according to the manufacturer's instructions.

### Preparation of nuclear extracts and electrophoretic mobility shift assays (EMSA)

Nuclear extracts of purified PBMCs were prepared according to a technique described by Trede et al. [27] and modified for our experimental setup [56]. The intensity of shifted bands was normalized to bands of unstimulated controls. Competition experiments with labeled and unlabeled mutated oligos of the cytokine promoter binding sites (see above) were carried out in order to prevent non-specific binding to nuclear proteins. For such experiments, an excess (5–50×) of unlabeled oligos was added to the nuclear extracts. For specificity of binding, supershift assays for NFκB with a combined p65/50 antibody were performed (data not shown). To exclude non-specific reactions, a 30-fold molar excess of Oct-1 DNA (unrelated DNA, Table 1) was used, which did not compete with specific binding.

### Immuno (Dot) blot

To determine whether the DNA binding proteins were related to NFκB and NFAT, dot blots using a SRC96D SNS minifold I dot blotter (Schleicher & Schüll, Dassel, Germany) were performed with the following positive controls and antibodies (all from Santa Cruz Biotechnology, Heidelberg, Germany): positive controls (10 ng/dot): NFκB p52 (80 kD) sc-4095WB, c-Rel (61 kD) sc-4030WB; Jurkat nuclear extract, PMA-stimulated; primary antibodies (1 μg/dot): anti-NFκB p50 (NLS) sc-114, anti-NFκB p65 (A) sc-109, anti-NFκB p52 (447) sc-848, a rabbit polyclonal IgG1 antiserum, anti-c-rel (N466) sc-272, anti-NFATc2 (M-20) sc-1151, anti-NFATc1 (K-18) sc-1149, two goat polyclonal IgG1 antisera; secondary antibodies (0.8 μg/dot): goat anti-rabbit IgG AP-conjugate sc-2007, and donkey anti-goat IgG AP-conjugate sc-2022. The nuclear proteins were blotted onto a nitrocellulose membrane (Bio-Rad). After blocking with PBS/3% BSA (Fluka, Deisenhofen, Germany), the blot was incubated overnight with the primary antibodies diluted 1:2000 in PBS/1% BSA, washed again and incubated with the alkaline phosphatase-conjugated secondary antibody (1:500 in PBS/1% BSA). The blot was developed using the Vectastain^® ^staining kit (Vector Laboratories Inc. Burlingame, CA, USA).

### Cytokine measurements

Supernatants of cell cultures were harvested 3, 6, 9, 12, 18, 24, 30, 36 and 48 hours after stimulation and stored at -80 °C until used and thawed only once. IFNγ, TNFα, IL-1 and IL-6 were determined using an ELISA from Bender Systems (Vienna, Austria), IL-1RA and IL-8 by an ELISA from R&D Systems (Wiesbaden-Nordenstadt, Germany), and TGFβ 1 using an ELISA as previously described [57]. Quantification of IL-2 as an indicator for the bioactivity of IL-1 was done as previously described [46]. Samples were diluted in the same buffer as that used for the standards. All cytokines were quantified using an ELISA plate reader (Anthos Labotec, Salzburg, Austria or Microplate Reader, BioRad). Cytokine amounts were all within the range of the standard curve. Only stimulation of PBMC observed in donors showing no spontaneous cytokine release was included in the statistics.

### Inhibition of transcription factors

To inhibit translocation and binding of transcription factors to the IL-1RA promoter, PBMC were incubated for 1 h with each of the following substances (concentrations were based upon 50% inhibition): 50 μg/ml of CAPE (caffeic-3,4-dihydroxycinnamic-acid-phenyl-ester), (Biomol, Hamburg, Germany); 400 ng/ml cyclosporin A (Calbiochem-Merck Biosciences, Bad Soden, Germany); 10 μg/ml cycloheximide (Santa Cruz Biotechnology) prior to incubation with GP. Band shifts were determined after 1 h incubation with GP as described before, including mutated oligos for NFIL-6 (TTA CAA CA*G *T*GG *ATT GCG ACA CTT AGT GGG) and NFATP2/3 (GGC GCA *G*AA AA*G G*TA AAA TAT TTA CTA TCT) binding sites within the IL-1RA promoter. PBMC RNA was isolated with an RNeasy kit (Qiagen, Germany) and messenger RNA was transcribed into cDNA with Reactin Ready First Strand kit (Biomol) and analysed for IL-1RA and GAPDH transcripts by PCR with a HotStart "sweet" PCR mastermix (Biomol) (95°C, 30 sec, 55°C, 30 sec, 72°C, 30 sec, 25 cycles for IL-1RA and 94°C for 30 sec, 50°C for 30 sec and 72°C for 45 sec, 25 cycles for GAPDH) following 18 h incubation with GP. IL-1RA protein was measured in the supernatant by ELISA after 24 h incubation with GP.

### Statistical analysis

The Kolmogorow-Smirnov test was used to evaluate, whether cytokine amounts and binding activities to transcription factors were normally distributed. Correlation coefficients and corresponding significances were analyzed by the Pearson test (normal distribution) or the Spearman test (non-normal distribution). To compare experimental data to a theoretical value (for example GP + TSST-1 vs GP/TSST-1 calc.) we calculated the sum of the individual effects and added these values into Fig. 1B, 3, 4, 5, and 6. Significances of differences between experimental data (for example GP vs LPS) as well as between experimental data and theoretical values (for example GP + TSST-1 vs GP/TSST-1 calc.) were analysed using the Student's t-test (normal distribution) or the Wilcoxon Signed-Rank test (non-normal distribution), (SPSS for Windows; SPSS Science Software, Erkrath, Germany).

## Abbreviations

LPS, lipopolysaccharide; TSST-1, toxic shock syndrome toxin 1; GP, glucan phosphate; PBMC, peripheral blood mononuclear cells; PMN, polymorphonuclear neutrophils; LRE, LPS-responsive element; NF, nuclear factor; IL, interleukin; BSA, bovine serum albumin; PBS, phosphate buffered saline; PMA, phorbol myristate acetate; ATP, adenosine triphosphate; SEM, standard error of the mean; n.s., non-significant (p > 0.05); CyA, cyclosporin A; CHX, cycloheximide.

## Authors' contributions

JL participated in the study design and coordination, developed the band shifts assays, performed the statistics and helped to draft the manuscript. UL carried out band shift assays and the dot blots. JH, CF and JMB carried out band shift assays and participated in the ELISA and PCR studies. HH designed the oligos for the analysis of the IL-1RA promoter. LR participated in the PCR experiments. PK performed the ELISA tests and the flow cytometry experiments. NW carried out the IL-1RA inhibition experiments. DLW conceived of the study and prepared the glucan phosphate. AM conceived of the study, participated in the study design and coordination and drafted the manuscript. All authors read and approved the final manuscript.
